# Model for Developing a Health-Related Quality of Life Questionnaire for Chronic Obstructive Pulmonary Disease

**DOI:** 10.1155/2018/6450962

**Published:** 2018-05-02

**Authors:** Nikolić Emilija, Nikolić Aleksandar, Stević Ruža, Brandmajer Tijana, Mićanović Veselin, Mašnić Jelena

**Affiliations:** ^1^Faculty of Medicine, University of Montenegro, 81000 Podgorica, Montenegro; ^2^D.o.o. “NCG Inzinjering”, 81000 Podgorica, Montenegro; ^3^Faculty of Medicine, University of Belgarde, 11000 Belgrade, Serbia; ^4^Faculty of Philosophy, University of Montenegro, 81000 Podgorica, Montenegro

## Abstract

**Introduction:**

The St. George's Respiratory Questionnaire (SGRQ), Modified Medical Research Council (mMRC) Dyspnea Scale, Hospital Anxiety and Depression Scale (HADS), and general health questionnaire (SF-36) are widely used for chronic respiratory diseases such as chronic obstructive pulmonary disease (COPD).

**Aim:**

We examined the reliability and validity of a modified questionnaire (MQ) to create a model for assessing the health-related quality of life (HRQOL) in COPD.

**Method:**

In total, 132 COPD patients completed the MQ. Lung function, smoking index, and exacerbation frequency were measured. Cronbach's *α* coefficient of correlation, standard deviation, and multifactorial nonlinear regression analysis were used to verify the internal validity of the MQ and to develop the mathematical model.

**Results:**

Female (63) patients had lesser airway obstruction than, and exacerbation frequency similar to that of, male patients. Exacerbation frequency significantly correlated with spirometry parameters in female patients. The MQ total score achieved high internal consistency (Cronbach's *α* = 0.89) and showed significant correlations with exacerbation frequency, smoking habit, and spirometry parameters in male patients (*p* < 0.005).

**Conclusion:**

The HRQOL questionnaire was shown to be a good indicator of the health status of COPD patients. The mathematical model easily and precisely confirmed the score of HRQOL questionnaire.

## 1. Introduction

Smoking is the most important cause of chronic obstructive pulmonary disease (COPD), although genes and environmental conditions, such as respiratory infections, air pollution, occupation, diet, and socioeconomic status, are also important aspects [1–3]. The General Guide for Chronic Obstructive Lung Disease (GOLD standard) defines treatment objectives for these patients, which include improving the physical activity and emotional state (quality of life, or QOL). An important clinical aim is preventing further progression and minimizing symptoms [4].

Anxiety and depression often present at later stages (III and IV) of COPD, which indicate a poorer prognosis [5]. The prevalence of depression in COPD patients is approximately 39% [6], and COPD patients have the highest risk of depression among chronic medical illnesses [7]. From a biological perspective, depression, anxiety, and COPD share similar pathological mechanisms. The proinflammatory cytokine interleukin (IL)-6 is increased in depression [8] and COPD [9]. Similarly, neuropeptide Y mediates anxiety and enhances the inflammatory response in bronchi [10]. From a psychological perspective, depression and anxiety predict the health-related quality of life (HRQOL) in COPD patients [11]. From a social perspective, both depression [12] and COPD [13] contribute to significant economic burdens.

The concept of HRQOL is present in medical research to the extent that recent studies have revealed that the measurement of HRQOL is crucial for clinical trials [14–16]. Questionnaires are the main instruments for measuring HRQOL. In addition to general questionnaires such as short form (SF-36), there are questionnaires for specific diseases [17]. While the St. George's Respiratory Questionnaire (SGRQ) for COPD has proven reliability and sensitivity to clinical changes in the disease, researchers now apply the revised version, the SGRQ-C [18]. Generic and disease-specific measures may capture complementary information, and it may be desirable to incorporate both types of measure in a study, depending on the goal of the study [19].

The Hospital Anxiety and Depression Scale (HADS) is a special questionnaire that identifies the emotional state of a patient. Developed by Zigmond and Snaith in 1983, its purpose is to provide clinicians with a tool for identifying and quantifying depression and anxiety in an acceptable, reliable, and easily applicable form in practice [7].

A recent study has shown that dyspnea can be a strong predictor of mental and physical outcome components in HRQOL questionnaires [20, 21]. Therefore, in this study, we investigated the correlation between the HRQOL of COPD patients and the following parameters: spirometry, smoking habit, and exacerbation frequency. Using multifactorial regression analysis, a mathematical model was developed, which can precisely determine the HRQOL score.

## 2. Materials and Methods

### 2.1. Study Population

A total of 132 consecutive COPD patients treated at the Mediterranean Health Centre Igalo and the Public Health Centre Herceg Novi between June 2016 and June 2017 were enrolled. The inclusion criteria included an existing COPD diagnosis and meeting the spirometry criteria for COPD (GOLD 2016) at the initial assessment. The exclusion criterion was a coexisting respiratory disease.

The study was conducted in accordance with the ethical standards of the Committee of Human Experimentation published by the Montenegrins Association of Physiotherapy (Ref. 01-28/08-2/2016), which was performed in accordance with the Declaration of Helsinki.

All patients provided written informed consent.

### 2.2. Experimental Measures

COPD patients were invited to complete review questionnaires, including items regarding MQ, smoking habits, exacerbation frequency, and pulmonary function test results, at 6-monthly intervals for 1 year.

The SGRQ is a self-administered questionnaire developed by Jones and colleagues in 1991, containing 50 items in three domains. The first 8 items cover the frequency and severity of respiratory symptoms as the symptom domain, the next 16 items concern limitations in activities because of the shortness of breath as the activity domain, and the last 26 items cover the approval, social, and psychological consequence of respiratory diseases as the impact domain. Its results are given as a score ranging from 0 (entirely satisfactory QOL) to 100 (maximally reduced QOL). The questionnaire has been validated for COPD and asthma and is used to assess the health status of other respiratory diseases, such as idiopathic pulmonary fibrosis, bronchiectasis, cystic fibrosis, and pulmonary tuberculosis [22].

We used the 36-Item Short Form Health Survey (SF-36) questionnaire, which contains 36 questions regarding the patient's general condition, including physical condition, general health, vitality, social functioning, emotions, mental health, activity, and pain. The number of points recorded in each scale of the questionnaire is transformed into standard values and calibrated on a unique scale with a theoretical minimum of 0 points, indicating poor general health, and a maximum of 100 points, indicating excellent general health. This scale has been used to study the general health status of patients with chronic respiratory diseases, such as COPD, asthma, and bronchiectasis [23].

We also used the Modified Medical Research Council (mMRC) Dyspnea Scale, which is a good indicator of the functional capacity of patients' lungs. Level 0 indicates no dyspnea, Level 1 indicates dyspnea on an incline, Level 2 indicates that the patient walks more slowly than his or her peers, Level 3 indicates that the patient has to stop after walking 100 m, and Level 4 indicates that the patient is unable to leave the house [24].

Finally, we used the HADS, which measures the emotional state of patients and their degree of anxiety and depression. It contains 10 declarations in which the respondent indicates the extent to which they relate to him. Based on the total score, it is determined whether the patient has mild (0–10), moderate (10–25), or severe (25–40) anxiety and depression level [7].

Because certain topics covered in these four administered questionnaires are repeated, a modified survey was developed and adapted to this research. The most important stage in dealing with modified questionnaires (MQs) is calculating the results, or scoring, which is conducted in the following order: (1) entering data, (2) checking the entered data and determining if all questions have been answered, (3) collecting the obtained results, (4) transforming the results into a new score, and (5) checking the previous procedure.

The following parameters of the pulmonary function test were measured: (a) forced expiratory volume in the first second (FEV1) pre- and postadministration of a bronchodilator, (b) forced vital capacity (FVC), and (c) peak expiratory flow (PEF).

### 2.3. Statistical Analysis

The free statistical software Cronbach's alpha (V.1.01) was used to verify the consistency (internal validity) of the modified questionnaire structure. A correlation of ≥0.7 was assumed to indicate that questions within a dimension are likely to measure the same construct.

Data analysis was performed using SPSS version 20.0 (IBM Corp), with a *p* value < 0.005 indicating statistical significance for correlations (mild, moderate, and highly; *r* = ±0.26 – ±1) among pulmonary function parameters, smoking habit, and MQ.

Multifactorial nonlinear regression analysis was performed to define the model that would mathematically express the general physical condition, HRQOL, and the emotional state of COPD patients on the basis of pulmonary function parameters, smoking habits, and exacerbation frequency.

## 3. Results

Of 132 patients examined, 63 female patients had less airway obstruction than men (Table 1) and a similar exacerbation frequency as that of men (mean, 1.956 ± 0.864 versus 2.0 ± 1.163, resp.). Other patient statistics of lung function and exacerbation are shown in Table 1.

Correlations between spirometry, smoking habit, exacerbation frequency, and the MQ score for female and male patients are shown in Table 2.

The MQ score highly correlated with exacerbation frequency in both sexes (*r* = 0.60; *r* = 0.80; *p* < 0.005), moderately correlated with spirometry parameters in males (MQ/FVC, *r* = −0.49; MQ/FEV1, *r* = −0.41; MQ/PEF, *r* = −0.41), and mildly correlated with smoking habits in females (MQ/smoking habits, *r* = −0.33).

Exacerbation frequency mildly correlated with spirometry parameters. The greatest impact was noted in FVC (female, exacerbation/FVC, *r* = −0.47; male, exacerbation/FVC, *r* = −0.48).

The results of verifying the authenticity of customized questionnaires are given in Table 3, which show high reliability. The Cronbach's *α* was 0.8944, which is approximately the maximum value of 1.

Out of all respondents, 25% stated that they had some contact with a psychiatrist, but further analysis of this data was not provided. Indeed, 22% of respondents said they had used psychiatric drugs. Psychiatric heredity was listed as positive in 7.5% of patients, anxiety in 86%, and a depressive syndrome in 63%.

### 3.1. Development of Mathematical Model

Using the multifactorial nonlinear regression analysis, a statistical model that determined the MQ score using spirometry parameters, exacerbation frequency, and smoking habits was developed. The results of statistical analysis are given in Table 4.

The model used the following definition:

(1) $$\begin{array}{c} Y=\exp\left(a\times x1+b\times x2+c\times x3+d\times x4+e\times x5+f\right), \end{array}$$

where *Y* is the score of the questionnaire; *x*1 is smoking habit; *x*2 is the FVC value; *x*3 is the FEV1 value; *x*4 is the PEF value; *x*5 is exacerbation frequency; and *a*, *b*, *c*, *d*, *e*, and *f* are variable regression values.

A basic assumption made during the process of developing the model is that the dependent variable *Y* (score) can be determined using independent variables, *x*1–*x*5 (smoking habit, exacerbation frequency, and spirometry parameters).

The adequacy of the selection of independent variables is confirmed by proving the null hypothesis, that is, the regression coefficient of independent variables is equal to 0. This hypothesis is examined using the parameter Prob (*t*), which is the probability that the value of the regression coefficient with the independent variable is 0. Thus, a Prob (*t*) value of 0 indicates that the null hypothesis is not true. The fact that the selection of independent parameters of the model was adequate proves that the value of Prob (*t*), for 50% of coefficients with independent variables, is 0 (Table 5).

Coefficient values for (*B*), (*C*), and (*D*) amount to Prob (*t*) values of 0.034, 0.098, and 0.032, respectively, and the probability that the values of independent variables (FVC, FEV1, and PEF) will be 0 is 3.84%, 9.8%, and 3.2%, respectively. This result negates the assumption that lung function parameters substantially influence the HRQOL score of patients.

The *t*-ratio is the ratio of the estimated parameter and standard deviation of the same. The higher the *t*-ratio value, the more significant is the influence of independent variable on the dependent variable. In our model, the *t*-ratio for the parameter (*E*) of 11.660 was the highest, indicating that the influence of the coefficient *x*5, that is, exacerbation frequency, was the highest (Table 5).

The rating of the obtained model is based on the coefficient of determination, *R*^2^ (0 ≤ *R*^2^ ≤ 1; *p*=0.05). In addition to *R*^2^, an adjusted coefficient of determination, which can be <0, is used because of the introduction of a new factor that has no essential significance for variable-ranking but increases the value of the coefficient of determination. In our model, the resulting value for the coefficient of determination was *R*^2^ = 0.6362592869 and the adjusted coefficient of determination was *R*^2^ = 0.6218251316. The obtained coefficient of determination values indicate that 63.62% or 62.18% of the variation in the obtained score of the questionnaire can be explained by the developed model.

The model can be considered successful because, in practice, all models with a coefficient of determination *R*^2^ ≥ 0.6 are accepted.

The final model was as follows:

(2) $$\begin{array}{c} \text{SCORE}=\exp\left(-0.14\times \text{smoking habit}-5.86\times \text{FVC}-4.44\times \text{FEV}1+2.38\times \text{PEF}+0.17\times \text{ exacerbation }+3.50\right), \end{array}$$

where SCORE is the total results of the MQ, and FVC, FEV1, and PEF are spirometry parameters measured.

## 4. Discussion

Obstructive lung disease has been the subject of numerous medical and epidemiological studies. Some authors have argued that COPD can be defined as a smoking disease, given that an exposure to smoke is the primary criterion in the definition of the disease. When assessing the risk factors for COPD, spirometry is an important parameter. Thus, it is necessary to carefully evaluate the definition of respiratory obstruction (FEV1/FVC < 0.7) and not to overestimate or underestimate COPD among the elderly and young populations [14].

In several studies, approximately 97.5% of respondents were male; therefore, COPD is considered to be a disease affecting the male population [11]. COPD is highly correlated with smoking behavior, and the ratio of male to female smokers was 10.9 : 1 in Taiwanese adults [17].

In our study, a large number of female smokers (74%) had COPD compared with their total number. The smoking habit negatively correlated with the MQ score in both sexes. Smoking habit noticeably changed the HRQOL in female patients, with a 25% lower HRQOL score than male patients. However, the physiological and neurological factors that contributed to the hypersensitivity of the female body and the bronchial tree, in particular tobacco smoke and harmful substances from cigarettes, are unclear. A biological mechanism that explains the relationship between the smoking index and the HRQOL score in COPD patients has been proposed. A previous study has found that the proinflammatory cytokine IL-6 increased as the COPD stage worsened in smokers [9]. Furthermore, an increase in IL-6 has been found in patients with depression [8], which might lead to poor HRQOL scores in COPD patients. In our study, the lowest FEV1 value for female smokers was 0.6 L and the largest was 3.8 L. In female nonsmokers, the FEV1 values ranged from 1.4 to 4 L. Male smokers with COPD had an FEV1 ranging from 0.7 L (min) to 5 L (max) and nonsmokers had an FEV1 ranging from 1.9 L (min) to 4.2 L (max). In addition, male smokers had a moderate change in the HRQOL scores.

Moreover, our assessment showed a mild correlation between exacerbation frequency and smoking index (Table 2), which confirms the aforementioned research.

An important indicator of COPD severity is the HRQOL assessed using a questionnaire. Pickard et al. have shown that SGRQ scores are associated with a greater statistical power to discriminate among the levels of COPD severity using relative efficiency (RE) ratios rather than generic measures of health. The SGRQ exhibited a strong correlation with clinically measured FEV1 (*r* = 0.43) [15].

In this study, the lung function parameter FEV1 positively correlated with the total MQ score (male MQ/FEV1, *r* = 0.41), which can be used as the relevant indicator of HRQOL. Specifically, in both female and male patients, FVC values moderately correlated with the total MQ score.

The GOLD standard 2016 defines COPD exacerbation as an acute clinical event characterized by the worsening of respiratory symptoms, which is different from usual variation in symptoms. In our research, exacerbation frequency significantly correlated with the MQ score, confirming the previously conducted finding that exacerbations in patients with COPD is related to the HRQOL score [25, 26]. Patients with COPD and comorbid depression have more exacerbations and a reduced HRQOL. Moreover, COPD is a risk factor for the development of depression, particularly in patients with increased disability. Among comorbidities, depression is one of the most frequent because even mild COPD patients have a more than fourfold increase in the prevalence of depression compared with controls [27, 28].

In our study, 22% of COPD patients used psychiatric drugs, anxiety was present in 36%, and a legacy of psychiatric heredity was positive in 7.5%.

In addition, a meta-analysis of the correlation between factors associated with disease-specific HRQOL, as measured by the SGRQ, among COPD patients indicated that dyspnea was a key factor of HRQOL [5].

This study provides a further confirmation of the associations among dyspnea, objective measures lung functioning, and HRQOL. The SGRQ total score declined across GOLD stages 1–4. HRQOL was worse among patients with higher dyspnea (mMRC levels, 2–4) than among those with lower dyspnea (mMRC level, 0-1).

However, the parameters of lung function do not completely measure the mental health, sleep, and fatigue of patients. Therefore, further research is needed on COPD in clinical practice in terms of additional measurements of the health condition of patients [29, 30].

We concluded that smoking, parameters of lung function, and exacerbation frequency can be used to assess HRQOL.

Using multifactorial nonlinear regression analysis, we developed a mathematical model that can precisely determine the HRQOL score. This model enabled us to determine the quality of life of patients in a quick, simple, and efficient way with minimal time and participation. We recommend the use of the model when assessing COPD patients in clinical practice because it precisely confirms the total HRQOL score.

Therefore, we can expect an escalation in the number of female patients with COPD, which was confirmed in our study. According to the World Health Organization, in the last 10 years, the number of female smokers has increased and this trend is ongoing. Montenegro tops the list of European countries with the highest number of women who actively smoke cigarettes.

We hope that our work will promote a wider exploration of the development of the HRQOL model for COPD patients in both clinical practice and research studies. This version of the HRQOL model could enhance the clinical usefulness and acceptability of routine health status assessment by clinicians, thereby saving time and materials.

The limitations of our study are as follows: (1) this study had a relatively small number of patients; (2) although the HADS is commonly used to assess depression and anxiety in other chronic medical disorders [12, 31], it was found to exhibit low diagnostic accuracy for depression in COPD patients [32] and biased when assessing the severity of depression in COPD patients [33]; and (3) this study did not assess passive smoking, which plays a key role in the worsening of respiratory symptoms [31].

## 5. Conclusion

The HRQOL questionnaire is a good indicator of the health status of COPD patients. Exacerbation frequency and smoking status significantly changed the HRQOL scores. The smoking index reduced the HRQOL by 25% in female patients compared with that in male patients. Using multifactorial nonlinear regression analysis, a mathematical model was developed by which the scoring of the HRQOL questionnaire can be obtained in a simple and easy way using spirometry parameters, smoking habits, and exacerbation frequency during the year. By applying the obtained model, the HRQOL score can be determined and the success of therapy can be predicted. We believe that our work will stimulate the further validation of the model by measuring HRQOL in larger studies as well as use with similar respiratory conditions such as asthma.

## Figures and Tables

**Table 1:** Descriptive statistics of lung function and exacerbation.

*Female*	FVC	FEV1	PEF	Exacerbation number
Maximum value	4.6	4.12	10.21	3
Minimum value	1.59	0.58	1.81	0
Range	3.01	3.54	8.4	3
Mean	3.363	2.325	5.652	1.956
Standard deviation	0.948	0.831	1.912	0.864

*Male*	FVC	FEV1	PEF	Exacerbation number

Maximum value	6.92	5.39	11.29	3
Minimum value	2.25	0.74	1.58	0
Range	4.67	4.65	9.71	3
Mean	4.307	3.001	6.455	2
Standard deviation	1.183	1.238	2.271	1.163

**Table 2:** Correlation between the parameters of spirometry, smoking, exacerbations, and the modified questionnaire score.

*Female*	Smoking	FVC	FEV1	PEF	Exacerbation	MQ score
Smoking	1	0.0895	−0.0958	−0.1988	0.0852	−0.3347
FVC	0.0895	1	0.7983	0.5674	−0.4705	−0.3960
FEV1	−0.0958	0.7983	1	0.8197	−0.4292	−0.2691
PEF	−0.1988	0.5674	0.8197	1	−0.4438	−0.2219
Exacerbation	0.0852	−0.4701	−0.4292	−0.4438	1	0.6015
Score	−0.3347	−0.3960	−0.1691	−0.1219	0.6015	1

*Male*	Smoking	FVC	FEV1	PEF	Exacerbation	Score

Smoking	1	0.0072	−0.0680	0.0673	0.2357	−0.0009
FVC	0.0072	1	0.8379	0.8556	−0.4899	−0.496
FEV1	−0.0680	0.8379	1	0.8942	−0.3661	−0.4142
PEF	0.0673	0.8558	0.8942	1	−0.3318	−0.4177
Exacerbation	0.2357	−0.4899	−0.3661	−0.3318	1	0.8013
Score	−0.0009	−0.4969	−0.4142	−0.4177	0.8013	1

**Table 3:** Cronbach's *α* coefficient for the five questionnaires conducted.

	Cronbach's *α*	Std. *α*	G6 (smc)	Average *R*
Total	0.8944	1	1	1
MQ	0.95	1	1	1
SGRQ	0.8312	1	1	1
mMRCDyspnea Scale	0.7674	1	1	1
SF-36 Scale	0.8204	1	1	1
HADS	0.8697	1	1	1

**Table 4:** Multifactorial nonlinear regression analysis values for model development.

Number of observations = 132
Nonlinear iteration limit = 250
Diverging nonlinear iteration limit = 10
Number of nonlinear iterations performed = 5
Residual tolerance = 0.0000000001
Sum of residuals = 2.4163
Average residual = 1.8305
Residual sum of squares (absolute) = 3806.8881
Residual sum of squares (relative) = 3806.8881
Standard error of the estimate = 5.4966
Coefficient of multiple determination (*R*^2^) = 0.6362
Proportion of variance explained = 63.6259%
Adjusted coefficient of multiple determination (*R*_a_^2^) = 0.6218251316

**Table 5:** Regression variables.

Variable	Value	Standard error	*t*-ratio	Prob (*t*)
*A*	−0.1482	2.7698	−5.351	0.0
*B*	−5.8662	0.0194	−0.301	0.0341
*C*	−4.4479	2.6682	−1.666	0.098
*D*	2.3828	1.1024	2.161	0.032
*E*	0.1752	1.5025	11.660	0.0
*F*	3.5046	6.8791	50.945	0.0

*A*, variable regression for *x*1, smoking habit; *B*, variable regression for *x*2, value of FVC; *C*, variable regression for *x*3, value of FEV1; *D*, variable regression for *x*4, value of PEF; *E*, variable regression for *x*5, exacerbation frequency; *t*-ratio, ratio of the estimated parameter and standard deviation of the same; Prob (*t*), probability that the value of the regression coefficient with the independent variable is 0.
